# Changes in Non-invasive Myocardial Stroke Work Related to Variation in Pacing Sites and Heart Rates in Adolescents

**DOI:** 10.1007/s00246-025-03798-5

**Published:** 2025-02-12

**Authors:** Shankar Baskar, Hieu T. Ta, David S. Spar, Richard J. Czosek, Nicholas J. Ollberding, Justin T. Tretter

**Affiliations:** 1 The Heart Institute, Cincinnati Children’s Hospital Medical Center, 3333 Burnet Avenue, MLC 2003, Cincinnati, OH 45229-3026 USA; 2 Department of Pediatric Cardiology, Cleveland Clinic Children’s, and the Heart, Vascular, and Thoracic Institute, Cleveland Clinic, Cleveland, OH USA; 3 Division of Biostatistics and Epidemiology, Cincinnati Children’s Hospital Medical Center, Cincinnati, OH USA; 4 Department of Pediatrics, University of Cincinnati College of Medicine, Cincinnati, OH USA

**Keywords:** Myocardial Work, Force–Frequency relationship, Pediatrics, Echocardiography, Pacing site

## Abstract

Non-invasive assessment of myocardial work is a newly described technique to assess myocardial energetics. This has not been previously studied to assess the effects of right ventricular pacing at different sites or at different heart rates in children. We aimed to study the effects of right ventricular apical, septal, and His bundle pacing on myocardial work along with the effects of increasing heart rate. This was a prospective pilot study performed on six patients with structurally normal hearts and function following an electrophysiology study. Global work index and global work efficiency was highest during His pacing and lowest during right ventricular apical pacing. The global constructive work, index, and efficiency were progressively worse with increasing heart rates. In this prospective pilot study, we demonstrated that myocardial work indices differ depending on myocardial activation pattern and at different heart rates in pediatric patients. Myocardial performance as assessed by myocardial work efficiency is worse when pacing is performed at the right ventricular apex when compared to His and right ventricular septal pacing. Myocardial performance can be affected by higher heart rates, following a work–frequency relationship.

## Introduction

Pediatric patients with congenital or acquired high-grade heart block are often dependent on chronic ventricular pacing. It is not uncommon for these patients to develop left ventricular (LV) dysfunction, which is thought to be secondary to electromechanical dyssynchrony caused by pacing [1–5]. Although LV epicardial apex or mid/lateral wall pacing demonstrates the best ejection fraction on follow-up, left ventricular lead placement is not always possible, and the ideal right ventricular (RV) endocardial site is unclear [6]. Furthermore, the assessment of ejection fraction is highly preload and afterload dependent, with alternative means of understanding ventricular contractility, such as myocardial deformation, being highly afterload dependent [7]. Since RV endocardial pacing remains the mainstay for long-term pacing in children of adequate size for transvenous pacing, the site of pacing becomes important. While acute invasive hemodynamic assessment has shown some benefit with RV septal pacing compared to RV apical pacing, long-term follow-up studies have demonstrated equivocal results [8–12].

Non-invasive assessment of myocardial work is a newly described and relatively load independent technique for evaluating myocardial energetics. This technique combines left ventricular global longitudinal strain (GLS), i.e., shortening or lengthening of myocardial fibers, with non-invasive systolic blood pressure as an estimation of left ventricular afterload to derive pressure-strain loops [13, 14].

The following myocardial work indices are derived from these pressure-strain loops:

- Global work index (GWI): the total amount of work derived from the area of the pressure-strain loop.
- Global constructive work (GCW): positive work performed in the form of myocardial shortening during systole and negative work in the form of myocardial lengthening performed during isovolumetric relaxation.
- Global-wasted work (GWW): negative work performed during systole with myocardial lengthening and positive work in the form of myocardial shortening performed during isovolumetric relaxation.
- Global work efficiency (GWE): the ratio of GCW over the sum of GCW and GWW.

Given these properties, myocardial work may help compare the efficiency of different sites of pacing.

Preliminary studies suggest that these non-invasive myocardial work indices can predict responders to cardiac re-synchronization therapy (CRT) [15–17]. However, the role of non-invasive myocardial work assessment has not been explored previously in right ventricular (RV) pacing in adults, and no studies to date have assessed this non-invasive metric during pacing in children. Additionally, myocardial work as measured by this non-invasive technique is a static measurement that provides insight into the myocardial energetics at a single baseline heart rate. This limitation is particularly relevant in pediatric populations, where higher baseline heart rates in those with heart failure may affect myocardial work efficiency.

We conducted a pilot study to determine the effect of site of pacing site and heart rate on myocardial work, as derived from non-invasive pressure-strain loops, in an adolescent population undergoing invasive electrophysiology testing.

## Methods

This was a prospective pilot study performed in patients undergoing electrophysiology study (EPS) and ablation at Cincinnati Children’s Hospital Medical Center. The study was approved by the Cincinnati Children’s Hospital Medical Center Institutional Review Board (IRB). Patients ≥ 12 years of age (this age cut-off allows a baseline heart rate and blood pressure comparable to the adult population on whom the non-invasive myocardial work indices were developed) and ≤ 18 years of age with structurally normal hearts and function who were scheduled to undergo an EPS were approached to partake in the study. Consent and parental permission and/or assent were obtained from patients, and assent was obtained from participants 15–18 years of age. Those who consented and went on to have a successful ablation of an accessory pathway or slow pathway modification were included in the study. Patients who had evidence of other forms of electrophysiologic pathology such as atrial tachycardia, ventricular tachycardia, or heart block during the study were excluded.

The study was conducted in the electrophysiology (EP) laboratory upon completion of the ablation portion of the study and during the institutional routine wait period (1 hour) prior to the end of the case, during which intermittent EP testing is performed for return of the ablated substrate.

The study was conducted with the following pacing sequence using the EP catheters already in place for the EPS, confirming catheter positions with electroanatomic mapping system, and waiting 30 seconds after each change in condition to allow myocardial accommodation. The following pacing sites and rates were obtained:

1. Right atrial Pacing: At incremental heart rates (100, 130, 160 bpm).
2. His Bundle pacing: 100 bpm.
3. Right ventricular apical (RVA) pacing: 100 bpm.
4. Right ventricular septal (RVS) pacing: 100 bpm.

Right atrial pacing was used to study myocardial work at increasing heart rates, while different RV sites including the HIS bundle region were used to study the effects of ventricular pacing at various sites. Using a Vivid GE portable echocardiography machine, we obtained 3-beat acquisitions of standard 2D apical 2-, 3-, and 4-chamber images for each separate pacing site and each paced heart rate. Brachial cuff blood pressure readings was obtained during each set of image acquisition. Post-processing was performed after the electrophysiology procedure on the EchoPAC Clinical Workstation to obtain speckle tracking echocardiography and subsequent myocardial work analysis by a single observer (JTT) as previously described [18].

We estimated mean values for myocardial work indices and variability during right atrial pacing (at incremental heart rates), His bundle (HIS) (baseline activation, fixed rate), RV apical (RVA, fixed rate), and septal (RVS, fixed rate) pacing during the above-noted heart rates. His and Ventricular pacing was performed without atrial pacing to avoid intrinsic conduction. Descriptive statistics including means and standard deviations were calculated for each myocardial work index under each condition. Box plots were created to visualize how work efficiency changes for different placements and pacing. Repeated measures one-way analysis of variance (ANOVA) was used to obtain estimates for the mean difference between conditions when averaged over all patients. Models were fit using linear mixed effects regression (LMER) estimated via restricted maximum likelihood and incorporated a random subject-specific intercept and a fixed effect term for condition. The Kenward–Roger approximation was used to obtain the model degrees of freedom, and Tukey’s multiple comparison correction was applied when estimating pairwise differences. Likelihood ratio tests fit via maximum likelihood were used to test for any difference across conditions (i.e., omnibus test). All analyses were performed using the R environment for statistical computing and graphics (version 4.1.1), and LMER models were fit using the LME4 package (version 1.1.27).

## Results

A total of six patients were included in this pilot study (Table 1). The participants were primarily female, with a mean age of 15 years and had similar heights, weights, and body surface areas. During the EP study, a left free wall accessory pathway (baseline pre-excitation in all patients with accessory pathways) was successfully ablated in five of the six patients and one patient underwent a successful slow pathway modification for atrioventricular nodal reentry tachycardia.

**Table 1:** Patient and Procedural Characteristics

Patient number	Age (years)	Gender	Height (Cm)	Weight	BSA	EP diagnosis prior to Ablation	EP intervention performed
1	18	F	162	72	1.76	Left Lateral AP	RF ablation
2	13	M	160	43	1.41	Left Lateral AP	RF ablation
3	18	F	166	62	1.69	AVNRT	RF slow pathway modificaiton
4	15	F	172	56	1.65	Left Lateral AP	RF ablation
5	15	F	161	62	1.65	Left Lateral AP	RF ablation
6	13	F	161	52	1.53	Left Antero-Lateral AP	RF ablation
Mean (± SD)	15 ± 2		164 ± 5	58 ± 10	1.62 (± 0.13)		

*AP* Accessory Pathway (baseline pre-excitation in all patients), *AVNRT* Atrio-Ventricular Nodal Re-entrant Tachycardia, *RF* Radio-Frequency

### Comparison of Different Sites: (Figs. 1D-H*, *2A)

**Fig. 1:**
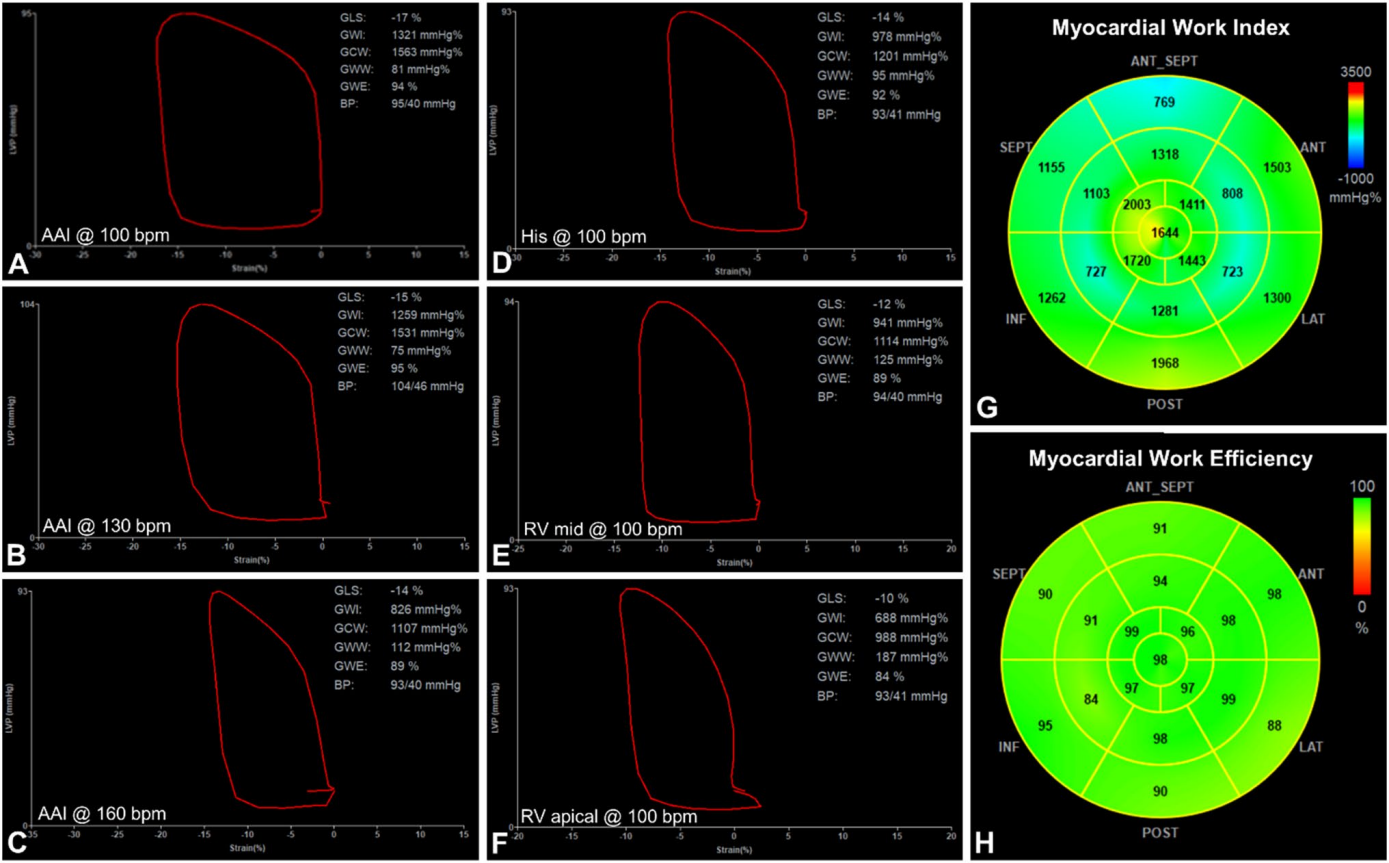
Example case of non-invasively derived pressure-strain loop and derived myocardial work indices from echocardiography. A-C AAI pacing was performed at increasing heart rates, demonstrating decreased global work index (GWI), global constructive work (GCW), and global work efficiency (GWE) with increasing global wasted work (GWW) at increased rates. D-F Similar trends where seen with differences in pacing site, from His bundle, to right ventricular (RV) mid-ventricle to RV apical pacing. Examples of regional derived myocardial work index (G) and myocardial work efficiency (H) are displayed from the same patient during AAI pacing at 100 bpm. BP blood pressure, GLS global longitudinal strain

**Fig. 2:**
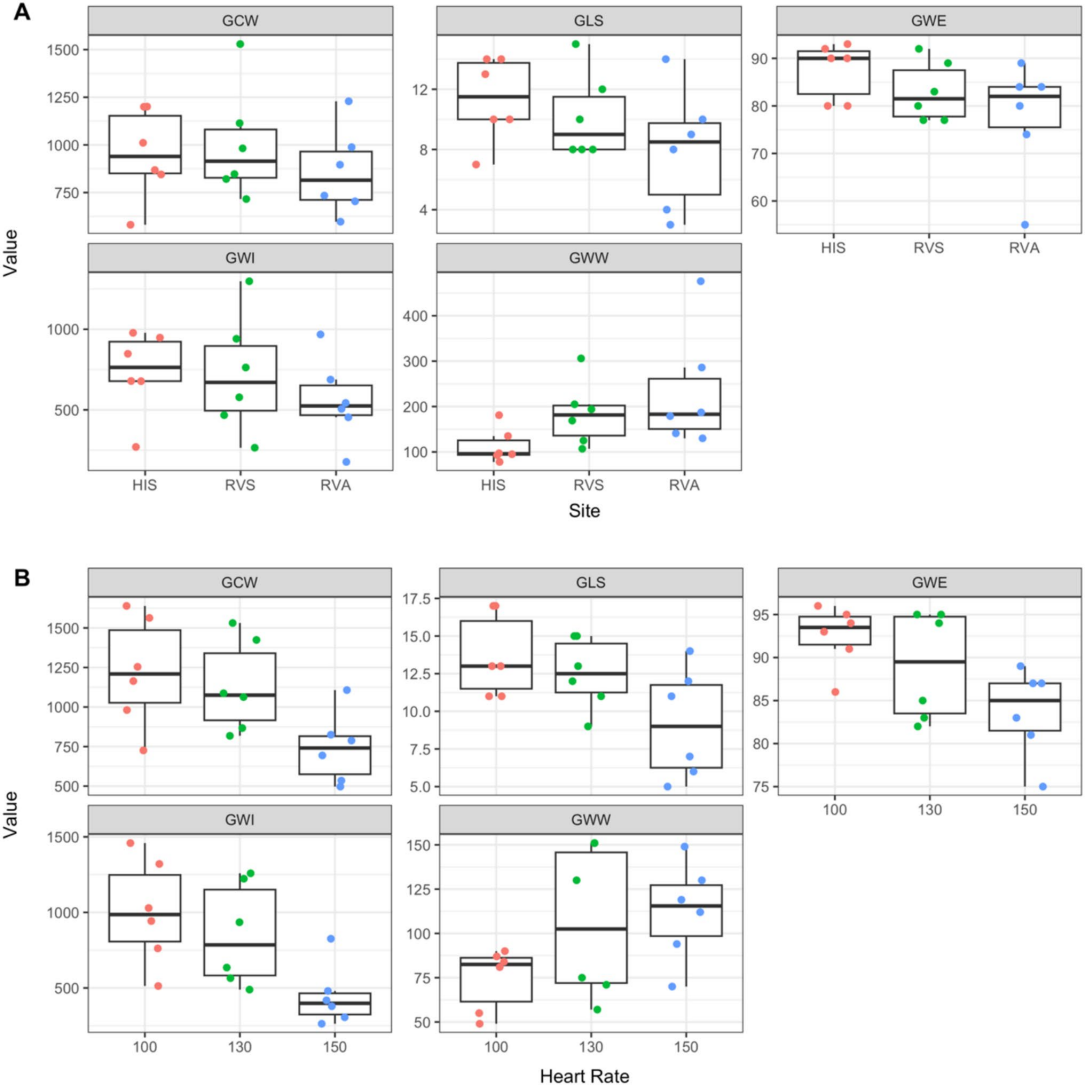
Myocardial work indices with pacing at different heart rates and different pacing site. A Myocardial work indices based on pacing at different sites within the right ventricles. B Myocardial work indices based on pacing at incremental heart rates from the atrium. Each dot represents an individual patient in both A & B. *GCW* global constructive work, *GLS* global longitudinal strain, *GWE* global work efficiency, *GWI* global work index, *GWW* global wasted work, *HIS* – His pacing, *LRT* likelihood ratio test, *RVA* RV apex pacing, RVS septal pacing

Based on the likelihood ratio tests, statistically significant differences were observed for measured indices at different sites even at this sample size (Table 2). The mean GLS was highest with HIS pacing and lowest with RVA pacing. This difference remained statistically significant when comparing each site separately (HIS-RVS, HIS-RVA, RVS-RVA). The GCW was highest with RVS pacing and lowest with RVA pacing and reached statistical significance only when comparing RVS to RVA pacing. GWW was highest with RVA pacing and lowest with HIS, with the difference between HIS and RVA being statistically significant. While GWI and GWE were highest during HIS pacing and lowest during RVA pacing, the differences in GWI between pacing sites were not statistically significant. GWE was significantly different only between HIS and RVA pacing.

**Table 2:** Mean differences in myocardial work indices according to pacing site

site	HIS	RVS	RVA	HIS–RVS	HIS–RVA	RVS–RVA	LRT
GCW	951 (238)	1001 (293)	858 (229)	b = – 50.5 (58.4), pval = 0.673	b = 93 (58.4), pval = 0.293	b = 143.5 (58.4), pval = 0.079	ChiSq = 5.8, df = 2, pval = 0.055
GLS	11.3 (2.8)	10.2 (2.9)	8.0 (4.0)	b = 1.2 (0.8), pval = 0.369	b = 3.3 (0.8), pval = 0.006	b = 2.2 (0.8), pval = 0.06	ChiSq = 11.9, df = 2, pval = 0.003
GWE	87.5 (5.9)	83 (6.3)	77.7 (12.2)	b = 4.5 (2.9), pval = 0.3	b = 9.8 (2.9), pval = 0.016	b = 5.3 (2.9), pval = 0.198	ChiSq = 9.4, df = 2, pval = 0.009
GWI	733 (261)	719 (368)	556 (262)	b = 14.8 (113), pval = 0.991	b = 177.5 (113), pval = 0.302	b = 162.7 (113), pval = 0.359	ChiSq = 3.2, df = 2, pval = 0.203
GWW	113 (38.2)	184 (70.8)	233 (131)	b = − 71.2 (39.9), pval = 0.224	b = − 120 (39.9), pval = 0.032	b = − 48.8 (39.9), pval = 0.467	ChiSq = 7.8, df = 2, pval = 0.02

Values are mean (*SD*) unless otherwise noted. P-values for pairwise contrasts obtained using Kenward-Roger approximation to the model degrees of freedom and Tukey's multiple comparison correction

*b* beta, *pval p*-value, *ChiSq* chi-square, *df* degrees of freedom, *LRT* likelihood ratio test, *RVS*–*RV* septum, *RVA*–*RV* apex

### Comparison of Different Rates: (Figs. 1A-C*, *2B)

There was a statistically significant difference among all measured indices other than the GWW when comparing all rates together based on likelihood ratio tests (Table 3). The GLS, GCW, GWI, and GWE all were consistently worse as the rates were increased. This was statistically significant when comparing higher heart rates (100–150, 130–150). The GWW showed a similar trend of worsening at higher heart rates, although the differences were not statistically significant.

**Table 3:** Mean differences in myocardial work indices according to atrial pacing rate

Pacing	100	130	150	100–130	100–150	130–150	LRT
GCW	1221 (346)	1132 (290)	741 (222)	b = 89.5 (77.7), pval = 0.506	b = 480.2 (77.7), pval = < 0.001	b = 390.7 (77.7), pval = 0.001	ChiSq = 20.1, df = 2, pval = < 0.001
GLS	13.7 (2.7)	12.5 (2.3)	9.2 (3.7)	b = 1.2 (0.8), pval = 0.339	b = 4.5 (0.8), pval = 0.001	b = 3.3 (0.8), pval = 0.004	ChiSq = 18.1, df = 2, pval = < 0.001
GWE	92.5 (3.6)	89.0 (6.3)	83.7 (5.2)	b = 3.5 (1.6), pval = 0.112	b = 8.8 (1.6), pval = 0.001	b = 5.3 (1.6), pval = 0.017	ChiSq = 17.3, df = 2, pval = < 0.001
GWI	1005 (350)	851 (338)	445 (202)	b = 153.5 (89.5), pval = 0.247	b = 559.2 (89.5), pval = < 0.001	b = 405.7 (89.5), pval = 0.003	ChiSq = 19.7, df = 2, pval = < 0.001
GWW	74.3 (17.7)	106 (42.9)	112 (27.7)	b = − 31.5 (17.8), pval = 0.229	b = − 38 (17.8), pval = 0.132	b = − 6.5 (17.8), pval = 0.93	ChiSq = 5.3, df = 2, pval = 0.072

Values are mean (*SD*) unless otherwise noted. P-values for pairwise contrasts obtained using Kenward-Roger approximation to the model degrees of freedom and Tukey's multiple comparison correction

*b* beta, *pval p*-value, *ChiSq* chi-square, *df* degrees of freedom, *LRT* likelihood ratio test

## Discussion

In this prospective pilot study, we demonstrated that myocardial work indices, as well as global longitudinal strain, differ depending on myocardial activation pattern and at different heart rates. In this patient cohort, myocardial performance as assessed by myocardial work, specifically GWE, is worse when pacing is performed at the RVA when compared to HIS or RVS pacing. Additionally, myocardial performance can be adversely affected by higher heart rates.

Chronic RVA pacing has been associated with adverse LV systolic function due to interventricular and intraventricular dyssynchrony both in the pediatric and adult population [19]. However, measurement of acute effects of differential pacing has not shown significant difference in ventricular function or hemodynamics in a prior study even though non-physiologic stimulation of the ventricle resulted in electromechanical dyssynchrony [9]. The present study demonstrates improved myocardial performance, as reflected by increased GLS and corresponding increases in GCW and GWE, during HIS pacing when compared to isolated RVA or RVS pacing. There was an overall trend toward RVS pacing being superior to RVA pacing, with RVS pacing being comparable to HIS pacing. RVS pacing close to the central ventricular components of the conduction system have been thought to narrow the QRS complex and produced improved LV synchrony. Although prior studies have shown mixed results, the current study findings might support improved myocardial efficiency with RVS pacing. If confirmed on larger studies, this might support a preferential RVS pacing over RVA pacing [11].

Similar to Santoro et al*.* [20], who investigated the impact of heart rate on myocardial work in an adult cohort of healthy patients, our cohort showed a trend toward increased GWW with associated decreased GWE with increasing heart rate. This relationship is likely explained by the force–frequency relationship and decreased peak force generated as previously demonstrated in prior patients with congenital heart disease after cardiopulmonary bypass [21]. Given the adverse effect of increased heart rates on GWE, studies are needed to confirm whether a heart rate corrected measure of myocardial work is needed to assess myocardial performance.

Lastly, even though HIS and RVS pacing, and pacing at a heart rate of 100 bpm, provided superior results, both GLS and GWE were lower compared to established normal values in similar aged adolescents. For example, HIS pacing at 100 bpm resulted in a GWE of 87.5 ± 5.9%, compared to 95.5 ± 1.1% reported for normal subjects with a comparative mean age [18]. This discrepancy may be secondary to the positive effects of atrial contraction on ventricular myocardial performance. These findings suggest that right-sided pacing or by extension isolated RV activation in conditions such as heart block or AV non-synchronous pacing might not fully restore the normal electromechanical state.

## Limitations

The study is limited by the small sample size, with the differences observed here requiring validation in additional patient cohorts. Additionally, this study was conducted under general anesthesia after an EPS, during which the myocardial conditions and measured blood pressure might not be comparable to baseline. Repeated induction of SVT during the EPS and myocardial memory from ventricular pre-excitation prior to ablation may also have influenced the measured indices. Although it would have been ideal to allow a longer duration between pacing conditions, this was not possible due to anesthetic time constraints. The changes in myocardial work indices observed with increasing atrial pacing rates may not directly reflect those seen with sinus tachycardia, such as in heart failure, as atrial pacing is associated with an increased atrioventricular delay, whereas sinus tachycardia typically results in a decreased delay. The use of isoproterenol to augment heart rates could be a valuable approach for future studies exploring similar dynamics. The use of isolated ventricular pacing to compare myocardial work at different pacing sites may not fully represent the dynamics of AV sequential pacing; future studies involving patients with complete heart block could provide further insights. QRS duration was not measured for different RV septal pacing sites. Although each patient served as their own control, the results may not be generalizable to all RVS sites. The lack of randomization in the pacing maneuvers might have introduced variability in the measurements, and future studies should consider randomization of the various pacing conditions.

While our group has previously reported good intra- and inter-observer variability in measuring myocardial work at resting heart rates in normal subjects and patients following heart transplantation, intra-observer, and inter-observer variability were not measured during the current study and could be a confounding factor in the measurements at higher heart rates [18, 22].

## Conclusion

In this pilot study, we have demonstrated that, based on relative load independent measures of left ventricular function, the myocardial work is lower for RVS and RVA pacing compared to HIS activation. Larger-scale studies are needed to confirm the trend of improved myocardial work indices with RVS when compared to RVA pacing and to determine whether RVS pacing is comparable to His bundle activation. The myocardial work–frequency relationship identified in this study carries important implications for translating normal values to patients and conditions with higher heart rates.

## Data Availability

No datasets were generated or analysed during the current study.

## Abbreviations

GCW: Global constructive work
GLS: Global longitudinal strain
GWE: Global work efficiency
GWI: Global work index
GWW: Global wasted work
RVA: RV apex pacing
RVS: Septal pacing
